# Cardioprotective effect of cedrol in an inflammation systemic model induced by lipopolysaccharide: Biochemical and histological verification

**DOI:** 10.34172/jcvtr.33112

**Published:** 2024-06-25

**Authors:** Seyed Hamidreza Rastegar-Moghaddam, Sabiheh Amirahmadi, Mahsan Akbarian, Matin Sharizina, Farimah Beheshti, Arezoo Rajabian, Mohammad Hosein Eshaghi Ghalibaf, Mohaddeseh Azimi, Maryam Mahmoudabady, Mahmoud Hosseini

**Affiliations:** ^1^Department of Anatomy and Cell Biology, Faculty of Medicine, Mashhad University of Medical Sciences, Mashhad, Iran; ^2^Psychiatry and Behavioral Sciences Research Center, Mashhad University of Medical Sciences, Mashhad, Iran; ^3^Applied Biomedical Research Center, Mashhad University of Medical Sciences, Mashhad, Iran; ^4^Department of Physiology, Faculty of Medicine, Mashhad University of Medical Sciences, Mashhad, Iran; ^5^Neuroscience Research Center, Torbat Heydariyeh University of Medical Sciences, Torbat Heydariyeh, Iran; ^6^Department of Physiology, School of Medicine, Torbat Heydariyeh University of Medical Sciences, Torbat Heydariyeh, Iran; ^7^Neuroscience Research Center, Mashhad University of Medical Sciences, Mashhad, Iran

**Keywords:** Lipopolysaccharide, Cedrol, Heart, Inflammation, Fibrosis, Oxidative stress

## Abstract

**Introduction:**

Evidence declared lipopolysaccharide (LPS) initiates inflammatory responses by stimulating the abandon of cytokines, which may perturb organ function. On the other side, it has been suggested Cedrol has potential properties, including anti-inflammatory and anti-oxidative activities. Herein, this study was done to assess the protective effect of Cedrol against LPS-associated heart damage.

**Methods:**

Thirty-five rats (200-250 g) were sorted into five groups, including control, LPS, LPS-Cedrol 7.5 mg/kg, LPS-Cedrol 15 mg/kg, and LPS-Cedrol 30 mg/kg groups. Cedrol was administrated through injected intra-peritoneally for two weeks. The heart tissues were removed and malondialdehyde (MDA) as a lipid peroxidation marker, superoxide dismutase (SOD), and catalase (CAT) as antioxidant markers were assessed. Furthermore, the interleukin (IL)-6 level in cardiac tissue was measured and Masson’s trichrome methods were employed to appraise cardiac inflammation and fibrosis, respectively.

**Results:**

Inflammation induced by LPS was significantly accompanied by myocardial fibrosis which was shown by Masson’s trichrome staining (*P*<0.001). In addition, LPS administration enhanced the MDA level while it diminished the activity of anti-oxidant markers such as CAT and SOD (*P*<0.001 for all cases). In the histological results, Cedrol improved LPS-induced inflammation and cardiac fibrosis (*P*<0.01 to *P*<0.001). Cedrol also enhanced CAT and SOD activities, whereas declined MDA level in the cardiac tissue (*P*<0.01 to *P*<0.001).

**Conclusion:**

The current findings proposed that the administration of Cedrol exerted a protective role in LPS-associated heart damage by reducing inflammation, cardiac fibrosis, and oxidative stress.

## Introduction

 Lipopolysaccharide (LPS) is an antigenic component of gram-negative bacteria cell walls, which is widely used in experimental studies to induce an inflammatory response.^1^ As an immunogenic agent, LPS may help the researchers study the mechanisms of inflammatory procedures.^1,2^ LPS increases the discharge of inflammatory cytokines including interleukin (IL)-6 through the activation of intracellular pathways related to toll-like receptors (TLR) such as nuclear factor kappa beta (NF-kβ).^3^ Activation of TLR in the heart by LPS can trigger significant pathological changes because of the actuation of the innate immune system.^4-6^ Oxidative stress has been proposed to be involved in the pathophysiologic processes of LPS-induced toxicity. Systemic inflammation caused by LPS is mainly associated with the occurrence of oxidative stress in the cardiovascular system.^7^ Besides, oxidative stress is one of the pivotal contributors to cardiac fibrosis in LPS-challenged animals.^8-10^ There is evidence for the impress of the TLR signaling pathway in the pathogenesis of cardiac fibrosis.^11^ Also, LPS exposure is thought to cause structural changes such as cardiac fibrosis through its effect on the renin-angiotensin system activity in the heart tissue.^12^ Cardiac fibrosis is a hallmark of maladaptive hypertrophy which is characterized by the adverse accumulation of collagens and other extracellular matrix (ECM) elements following the activity of fibroblasts. In addition, cardiac fibrosis is one of the pivotal factors for heart failure that leads to diastolic dysfunction and it is associated with disability and even death in patients.^14^

 Medicinal plants have been used as important sources of anti-inflammatory and antioxidant agents for many years. Numerous kinds of research are ongoing to investigate the therapeutic potential of their various compounds in pathological situations such as cardiac fibrosis.^15,16^ Cedrol, isolated from Juniperus chinensis, is a natural bioactive sesquiterpene with many medicinal consequences including anti-inflammatory, antioxidant, and anti-cancer activity.^17^ In an experimental study conducted on a rat model of arthritis, administration of Cedrol exerted its therapeutic effects by modulating serum oxidative stress markers.^18^ As mentioned above, the administration of LPS can cause structural changes such as fibrosis in the cardiovascular system. On the other hand, no research has been done on the cardioprotective and anti-fibrotic properties of Cedrol. To this end, based on the anti-inflammatory and antioxidant outcomes of Cedrol, this research was designed to find out the potential for Cedrol to attenuate LPS-induced inflammation, oxidative stress, and fibrosis in the cardiac tissue.

## Material and methods

###  Chemicals and reagents

 Cedrol was purchased from Tinab Shimi Khavarmianeh Company (Mashhad, Khorasan Province, Iran), LPS was obtained from Sigma Company (USA), and the IL-6 assay kit was purchased from Karmania, Kerman, Iran.

###  Animals and Ethical Statement 

 Thirty-five male Wistar rats were kept in a standard condition with a 12:12 light-dark cycle, a regulated temperature at 22-24°C, and 40 -60% humidity with free access to food and water. All animal experimentations were approved by the Research Ethical Committee of Mashhad University of Medical Sciences under the Ethical Committee code (IR.MUMS.REC.1402.038).

###  Experimental design

**Figure 1:**
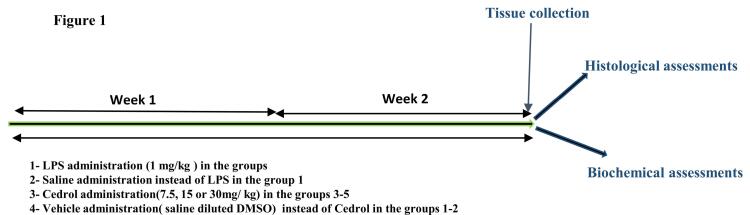
A diagram which shows the treatments and period of experiments

 G power software was utilized to estimate the sample size. Considering α = 0.05, power = 0.80, and employing the mean of malondialdehyde (MDA) concentration (max = 26, min = 16, and SD = 6 based on the prior studies), the sample size was computed to be 35. Animals were separated into 5 groups (n = 7/each group). Control group: the rats were given the vehicle, LPS group: 1 mg /kg of LPS was injected intra-peritoneally, Cedrol treated groups in which 7.5, 15 and 30 mg/kg Cedrol was injected intra-peritoneally before LPS infusion.^19^ The previous studies illustrated that repeated administration of LPS for 2 weeks is followed by heart tissue damage.^7,20^ The previous studies in which Cedrol was shown to have anti-inflammatory properties were considered to choosing the doses of Cedrol.^18,21^ Besides, some investigations were also considered for the method of administration of Cedrol.^22,23^ The injection of vehicle, LPS, and also the mixture of Cedrol with LPS was done during 2 weeks of the study. Cedrol was dissolved in the normal saline plus dimethyl sulfoxide (DMSO) and LPS was dissolved in the normal saline. After completing the prescription, the rats were humanely killed under anesthesia using ketamine-xylazine. A small piece of the cardiac tissue was homogenized in a cold phosphate buffer solution using a homogenizer. A 10 % solution (W/V) was prepared, and centrifuged. The supernatants were used for further analysis. Another part of the heart was washed with ice-cold saline and then immersed in buffered formalin 10% for histopathological evaluation (Figure 1).

###  Determination of antioxidant markers concentrations in cardiac tissue

 In the supernatants of heart homogenates, oxidative stress markers were determined based on the protocol of previous studies.

 In this study, the MDA level of the heart tissue was estimated as a profile of lipid peroxidation. Determination of the level of MDA was carried out using a mixture solution of trichloroacetic acid (TCA), thiobarbituric (TBA), and hydrochloric acid (HCL). The compounds were purchased from Sigma Aldrich Company, United States. In this colorimetric process, a red complex with a peak absorbance of 532 nm emerged when TBA responded with MDA.^24^

 The catalase (CAT) and superoxide dismutase (SOD) enzyme activity as well as the total thiol level of the heart tissue were investigated as indicators of the antioxidant capacity of the heart tissue. The level of total thiol was evaluated in the existence of 5,5′- dithiobis2-nitrobenzoic acid (DTNB, Sigma Aldrich Company, United States) as a reagent. After adding DTNB, the yellow mix with an absorbance pinnacle of 412 nm emerged when DTNB reacted with thiol levels.^25^ According to the approach of Aebi, the CAT activity was specified as the micromoles of the hydrogen peroxide detached, and the high level of CAT caused a decline in absorbance.^26^ In order to measure SOD enzyme activity, the superoxide resulting from pyrogallol auto-oxidation converted tetrazolium into red formazan with absorbance at 560 nm. The procedure was as earlier expressed.^27^

###  Inflammation assessment in cardiac tissue

 The interleukin-6 ELISA kit for rats was operated on to determine inflammation markers in the heart tissues matching the manufacturer’s teaching (Karmania, Pars Gene, Iran).

###  Masson’s trichrome staining and histopathological assessment 

 Cardiac tissue fix was performed through 10% formalin for 72 hours. Then, they were dehumidified alcohol, transparented in xylene, and entrenched in paraffin. Cut coronal serial sections by 5μm thick were carried out operating a microtome. Ultimately, cardiac slices were dyed with Masson’s trichrome and studied under light microscopy. In this staining, heart fibrosis was characterized by the collagen precipitate (blue-dyed) and an ample excess of connective tissue around the vessels. In summary, the samples via xylene and declining graded ethanol (100%, 90%, and 70%) were deparaffinized and rehydrated, respectively. Afterward, they were dipped in distilled water and located in Hematoxylin Weigert for 10 minutes. The next steps included washing with distilled water, immersing in fuchsine acid (30 seconds), and phosphotungstic-phosphomolybdic acid solution, respectively. Then, the samples were dyed with an aniline blue solution for 15-20 minutes. Ultimately, to determine tissue fibrosis, Image J software was used. The average staining intensity was calculated and changed to an optical density (OD) as previously procedure.^28^ The experimenter was blind to the treatments of the groups.

###  Statistical analysis

 The normalcy of the data was analyzed utilizing the Kolmogorov-Smirnov test. The findings of this research were submitted as mean ± SEM and GraphPad Prism 8.0 was employed to conduct statistical examinations. The significance between groups was specified using one-way ANOVA and post hoc test. *P* values < 0.05 were deemed statistically significant.

## Results

###  Inflammation 

**Figure 2:**
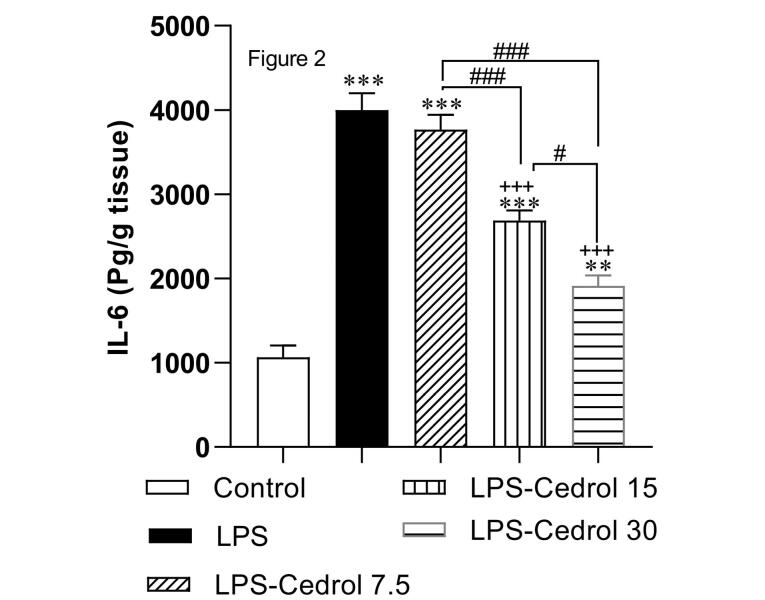
Cedrol administration reduced heart tissue inflammation induced by LPS. Comparison of IL-6 level in the heart among different groups. Data was expressed as Mean ± SEM. ** *P* < 0.01 and ***P < 0.001 compared to the control group, ^+ + +^*P* < 0.001 compared to the LPS group, ^#^*P* < 0.05 and ^###^*P* < 0.001 comparison between different doses of Cedrol

 To confirm LPS-induced inflammation, the interleukin-6 level was measured. As shown in Figure 2, the IL-6 level was markedly increased in the LPS group compared with the control group (*P* < 0.001). The comparison between the Cedrol-treated groups with the control group revealed that the IL-6 value was markedly higher than compared to the control group (*P* < 0.001, *P* < 0.001, and *P* < 0.01 for LPS - Cedrol 7.5, LPS - Cedrol 15 and LPS - Cedrol 30). However, Cedrol with doses 15 and 30 mg/kg induced a remarkable decrement in IL-6 level compared with the LPS group (both *P* < 0.001). Also, the IL-6 concentration in the LPS-Cedrol 30 group was remarkably lower than in the LPS-Cedrol 15 and LPS-Cedrol 7.5 groups (*P* < 0.05 and *P* < 0.001, respectively). It was also shown that treatment with 15 mg/ kg Cedrol was more influential than 7.5 mg/ kg (*P* < 0.001).

###  Oxidative stress 

**Figure 3:**
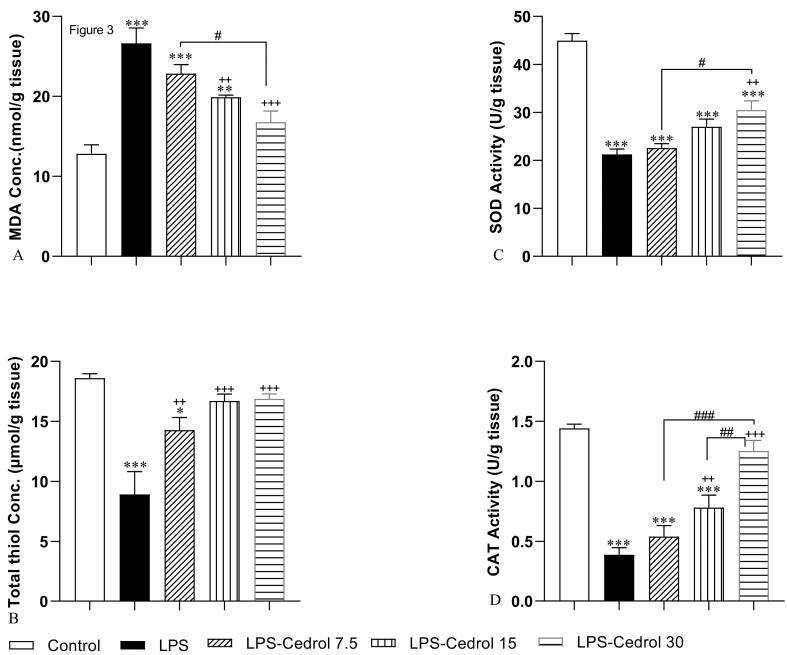
Cedrol administration reduced MDA and increased total thiol concentration and SOD and CAT activities in LPS-induced heart damage. Comparison of MDA level (A) and total thiol concentration (B) and SOD (C) and CAT activities (D) in the heart among different groups. Data was expressed as Mean ± SEM. *P < 0.05, **P < 0.01 and ***P < 0.001 compared to the control group. ^+ +^*P* < 0.01 and ^+ + +^*P* < 0.001 compared to the LPS group, ^###^*P* < 0.001, ^##^*P* < 0.01, and ^#^*P* < 0.05 comparison between different doses of Cedrol

 According to Figure 3 (A), injections of LPS markedly augmented the MDA level in the heart tissue (*P* < 0.001). Treatment with 15 and 30 mg/kg of Cedrol markedly alleviated the level of this index of oxidative stress in heart tissue (*P* < 0.01 and *P* < 0.001 respectively, compared to the LPS group), while this reduction was not considerable in the LPS - Cedrol 7.5 group. The level of MDA in both LPS-Cedrol 7.5 and LPS-Cedrol 15 was markedly higher than the control group (*P* < 0.01 and *P* < 0.001). The comparison of the Cedrol-treated groups shows that the amount of MDA reduction in the LPS-Cedrol 30 group was more than the LPS-Cedrol 7.5 group (*P* < 0.05), whereas there was no remarkable difference between the LPS-Cedrol 15 and LPS-Cedrol 30 groups.

 As shown in part B of Figure 3, LPS significantly reduced the total thiol amount of the heart samples (*P* < 0.001 compared to the control rats). However, treatment with Cedrol markedly attenuated the impacts of LPS on total thiol in all treatment groups (*P* < 0.01, *P* < 0.001, *P* < 0.001 for LPS-Cedrol 7.5, LPS-Cedrol 15 and LPS-Cedrol 30 compared to the LPS group). In addition, the graph shows a lower level in the total thiols in the heart of the LPS-Cedrol 7.5 group than the control group (*P* < 0.05). Also, the comparison between the treatment groups did not show a significant difference between the Cedrol-treated groups.

 The results of Figure 3 (C) indicated a reduced level of SOD activity in the heart tissue of the LPS-challenged group (*P* < 0.001 compared to the control group). In all Cedrol treated groups, the activity of SOD was lower than the control group (*P* < 0.001). Interestingly, only in the group receiving the high dose of Cedrol (30 mg/kg), a remarkable difference in the enhancement of SOD activity was observed in comparison with the LPS-challenged group (*P* < 0.01). The comparison of Cedrol-treated groups illustrated a remarkable difference in the SOD activity between the LPS-Cedrol 7.5 and LPS-Cedrol 30 groups (*P* < 0.05).

 The analysis of the results of Figure 3 (D) indicates that the administration of LPS has substantially reduced the activity of CAT in the heart tissue (*P* < 0.001 compared to the control group). However, treatment of animals with middle and high doses of Cedrol caused a significant increase in CAT activity (*P* < 0.01 and *P* < 0.001 respectively, compared to the LPS group). The activity of CAT in the LPS-Cedrol 7.5 and LPS-Cedrol 15 groups was lower than the control group (*P* < 0.001). A higher level of CAT activity was seen in the heart tissue of the animals treated with the highest dose of Cedrol than in the rats treated with other doses (*P* < 0.01 and *P* < 0.001, compared to the LPS-Cedrol 15 and LPS-Cedrol 7.5 groups respectively).

###  Fibrosis

**Figure 4:**
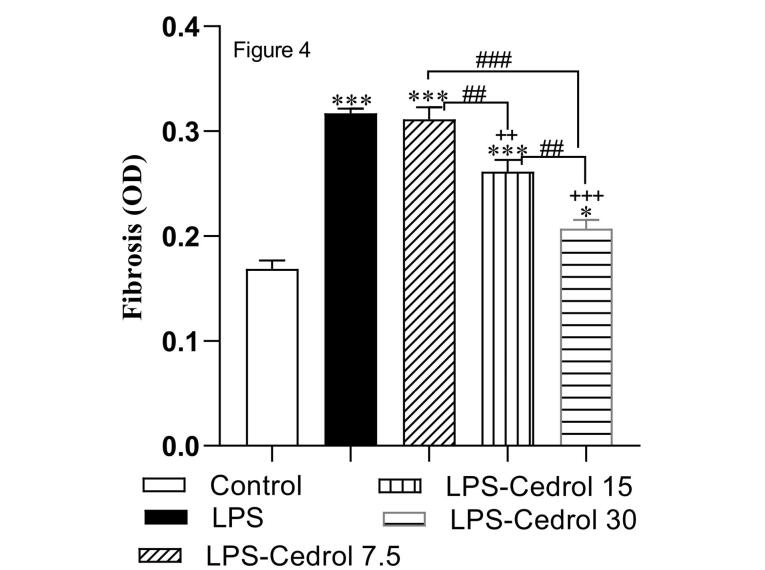
Cedrol administration reversed LPS-induced heart fibrosis. Comparison of histopathological results of fibrosis in the heart tissue between the different groups. Data was expressed as Mean ± SEM. ***P < 0.001 and *P < 0.05 compared to the control group. ^+ +^*P* < 0.01 and ^+ + +^*P* < 0.001 compared to the LPS group, ^#^*P* < 0.05 and ^###^*P* < 0.001, ^##^*P* < 0.01 comparison between different doses of Cedrol

**Figure 5:**
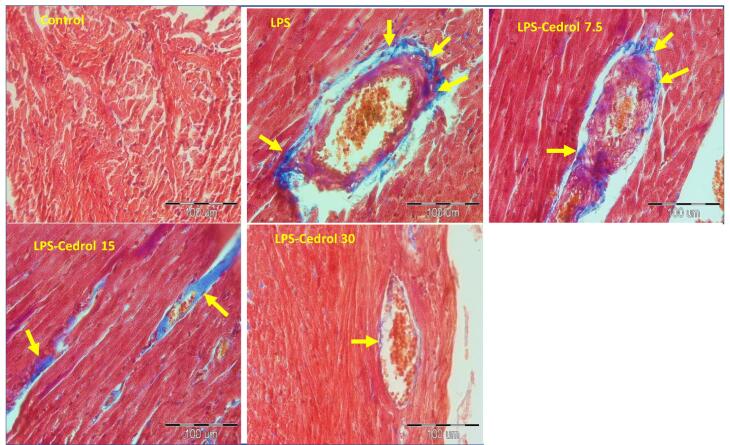
The photomicrographs show fibrosis in the heart tissue. The tissues were stained using Masson’s trichrome method. The blue color demonstrates collagen and fibrosis (yellow arrows) (Scale bar = 100 µm)

 The results of cardiac fibrosis are provided in Figures 4 and 5. The results of Masson’s trichrome staining represent significant cardiac fibrosis of the LPS-treated rats (*P* < 0.001 compared to the control rats). The comparison of the Cedrol treated groups with the control group in the index of fibrosis showed that their values were significantly higher than the control group (*P* < 0.05 and *P* < 0.001). Notably, treatment with Cedrol at 15 and 30 mg/kg doses substantially diminished cardiac fibrosis compared to the LPS group (*P* < 0.01 and *P* < 0.001). The statistical analysis showed a lower level of cardiac fibrosis in the LPS-Cedrol 30 group compared to the other treated groups (*P* < 0.01 and *P* < 0.001 for LPS-Cedrol 15 and LPS-Cedrol 7.5, respectively). Also, the fibrosis in the heart tissue of LPS-Cedrol 15 group was markedly lower than in the LPS-Cedrol 7.5 group (*P* < 0.01).

## Discussion

 The results of the present work showed that Cedrol attenuated fibrosis, oxidative stress, and IL-6 in the cardiac tissue of LPS-injected rats. In the present research, repeated LPS injection was followed by an inflammation response, which was characterized by the enhancement of IL-6 in the cardiac tissue. This result of the current study was in line with previous reports in which LPS increased pro-inflammatory factors in heart tissue.^29,30^ Inflammatory reaction as a pathological procedure has a strong role in the creation of cardiac damage.^31,32^ Following the recognition of LPS as a natural ligand by TLR4, activation of intracellular pathways such as NF-KB leads to the expression of target genes (e.g. IL-6). During inflammation, cytokines exudation in cardiac myocytes contributes to the infiltration of neutrophils into the myocardium, which may aggravate apoptosis and necrosis of cardiomyocytes.^33,34^

 In our study, LPS administration was followed by remarkable oxidative injury in the heart, as documented by a raised level of lipid peroxidation marker with a concomitant decline in endogenous antioxidant levels such as SOD, CAT, and total thiol. MDA affects cells through changes in cell membrane attributes, such as permeability and fluidity, as well as the velocity of protein degradation.^35^ Also, SOD can act as an antioxidant and reactive oxygen species (ROS) scavenger,^36^ and CAT may diminish ROS generation by degrading H2O2 into oxygen and water.^37^ In addition, thiol groups are compounds containing sulfhydryl group (‐SH) which can destroy ROS and other free radicals and act as a member of the antioxidant cascade.^38^ In line with the findings of our study, the capability of LPS for induction of oxidative damage in cardiac tissues was previously studied in rats.^39^ LPS can trigger oxidative injury by stimulating the production of free radicals and their accumulation in the tissues.^40^ Activation of mitogen-activated protein kinase (MAPK) and NF-κB transduction passages has been introduced as the mechanism(s) of the later-mentioned effects of LPS.^41^ Inflammation and oxidative stress and are two interdependent biological phenomena. Mitochondrial ROS can enhance the response to inflammatory factors. Beyond this, unrestricted ROS production at inflammatory sites accompanies oxidative stress-induced damage to mitochondria.^42^ The evidence of previous studies shows that LPS can affect the interaction of oxidative stress and inflammation. Exposure to LPS usually triggers the production of the pro-inflammatory mediators which may lead to the intemperate production of free radicals resulting in oxidative stress.^43-45^

 The result of our study showed that LPS injection caused fibrotic destruction in the cardiac tissue of rats, which is compliant with the observations of previous research.^8^ Interstitial fibrosis is a dangerous pathological condition that affects the ventricular wall of the heart and cardiac compliance, eventually leading to cardiac dysfunction. It should be noted that inflammation and fibrosis of cardiac tissue occurred together in our study. Therefore, IL-6 probably played a special role in the pathogenesis of fibrotic damage which was seen in the present study. Along with the mentioned hypothesis, previous studies have investigated the role of IL-6 as a pro-inflammatory mediator in the process of cardiac fibrosis. Due to the presence of IL-6 receptors in adult cardiac fibroblasts,^47^ IL-6 applications can promote collagen production, proliferation, and phenotypic conversion to myofibroblasts of cardiac fibroblasts in experimental studies.^48,49^ Furthermore, in IL-6 knockout diabetic mice, cardiac interstitial fibrosis was alleviated by modulating transforming growth factor beta 1 (TGFβ1) and miR-29 pathways.^50^ In the current research, Cedrol attenuated cardiac inflammation which was presented by a decrease in the IL-6 concentration in the heart tissue of LPS-Cedrol 15 and LPS-Cedrol 30 groups (Figure 2). Considering the evidence described above and the present study’s results, it looks like Cedrol prevented IL-6 overproduction and tissue inflammation which attenuated cardiac fibrosis. The anti-inflammatory consequences of Cedrol have been suggested in later investigations. In rats with rheumatoid arthritis, Cedrol administration reduced the secretion of pro-inflammatory cytokines and inflammation-related mediators through inhibition of phosphorylated-JAK3 protein.^21^ In another rat model of arthritis, treatment with Cedrol revealed a potent anti-inflammatory impact.^18^ In an in-vitro study, Cedrol significantly reduced inflammatory response in IL-1β-treated chondrocytes through promoting miR-542-5p expression.^51^ Moreover, Cedrol reduced the inflammatory response in fibroblast-like synoviocytes treated with LPS.^52^

 In addition, oxidative damage in our study was probably involved in the occurrence of cardiac fibrosis. Oxidative stress is an important regulator of cardiac interstitial fibrosis. For example, the result of a study showed that the ROS derived from Nox4 enzyme activity mediates TGF-β1-induced conversion of fibroblasts to pathological myofibroblasts by regulating Smad 2/3 activation.^53^ Also, in obese rats, mitochondrial oxidative stress induced cardiac fibrosis through increasing transthyretin (TTR) protein.^54^ Therefore, treatment with antioxidants can inhibit fibrotic damage in heart tissue by suppressing oxidative damage.^55,56^ The results of the present study showed that Cedrol attenuated MDA and improved SOD, CAT, and thiol (Figure 3). Therefore it seems that Cedrol prevented oxidative stress in the heart tissue which attenuated cardiac fibrosis.

 In the present study, treatment with Cedrol significantly reduced fibrotic damage in heart tissue (Figure 4 and 5). So far, no study has investigated the therapeutic effects of Cedrol on cardiac fibrosis, but the anti-fibrotic effect of other members of the sesquiterpene family has been reported in previous studies. It was previously demonstrated that the sesquiterpene compound could attenuate myocardial fibrosis by suppressing the TGF-β1/Smad signaling pathway in a mice model of myocardial infarction.^57^ It was also revealed that administration of another sesquiterpene compound ameliorated bleomycin-incited lung fibrosis by quelling TGF-β-induced fibroblast to myofibroblast differentiation.^58^

 Overall, for the first time, the results of our study show that the administration of Cedrol reduces the occurrence of cardiac fibrosis in rats challenged with LPS through the suppression of inflammation and oxidative stress. Our study had some limitations; we used a low dose of LPS, which only causes systemic inflammation, and to study the effect of specific clinical situations caused by severe infections and sepsis, future studies have to be done to evaluate the therapeutic effect of Cedrol after injection of higher doses of LPS. The current study attributed the anti-fibrosis effects of Cedrol to its antioxidant and anti-inflammatory features, but the molecular-cellular mechanisms involved in these effects were not investigated, which should be considered in future studies. Our results propose that administering Cedrol can reduce the cardiovascular complications caused by inflammation. In light of this, the cardioprotective role of Cedrol in such disturbance can be regarded as a complement to other usual treatments. Nonetheless, a better perception of the procedures underlying Cedrol’s functions entails further research in the future.

## Conclusion

 Our results support that the administration of Cedrol has a protective impact on LPS-associated cardiac fibrosis by reducing inflammation and averting oxidative stress.

## Acknowledgments

 This study was financially supported by Mashhad University of Medical Sciences( 4011830).

## Competing Interests

 The authors asserted no conflicts of interest relating to the research and/or publication of this work.

## Ethical Approval

 The experiments were approved by the Research Ethical Committee of Mashhad University of Medical Sciences (IR.MUMS.REC.1402.038).
